# Hyperacute assessment of vertigo in suspected stroke

**DOI:** 10.3389/fstro.2023.1267251

**Published:** 2023-09-19

**Authors:** Stacy Morrow, Nehzat Koohi, Diego Kaski

**Affiliations:** ^1^Global Product Management, Natus, Taastrup, Denmark; ^2^SENSE Research Unit, Department of Clinical and Movement Neurosciences, University College London, London, United Kingdom

**Keywords:** hyperacute, vertigo, stroke, dizziness, education, assessments, telemedicine, HINTS

## Abstract

The management of patients with acute vertigo is most challenging in the hyperacute phase, both due to the complexity of vertigo as a symptom, the range of possible causes, and the lack of training in neuro-otology for non-specialists. Perhaps of greatest relevance is differentiating between peripheral (usually benign, e.g., inner ear) causes and central (potentially more sinister, e.g., stroke) causes. Several diagnostic algorithms have been introduced to help detect stroke in patients with acute vertigo. However, these algorithms have been largely validated in patients with an acute vestibular syndrome (with nystagmus) for whom symptoms have been present for a minimum of 24 h. The most challenging period within the diagnostic process is the hyperacute phase that determines triage and treatment, but where none of the established algorithms have been validated. In this review, we specifically describe practical implementation considerations for evaluating patients with hyperacute vertigo, including the timing of diagnostic testing within the emergency department pathway, resource availability, and pitfalls associated with current practices.

## 1. Introduction

### 1.1. Diagnostic errors in the emergency department

Suspected acute ischemic stroke is an emergency requiring hyperacute assessment to facilitate proficient management to reduce morbidity and mortality. A systematic review completed on the diagnostic errors in the emergency department (ED) found that stroke, the top serious harm-producing disease, is missed 17% of the time and dizziness or vertigo increases the odds of misdiagnosis 14-fold over motor symptoms (those with isolated dizziness and vertigo are missed initially 40% of the time) (Newman-Toker et al., 2022). Patients with stroke presenting with acute vertigo–so-called central acute vestibular syndrome–that affects the posterior circulation of the brain, often seek medical attention late or are inappropriately triaged, because they do not experience “typical” stroke symptoms (facial droop, limb weakness or speech impairment) (Banerjee et al., 2018). Patients tend to be younger than those with anterior circulation strokes, and such posterior circulation strokes carry greater mortality due to malignant transformation causing swelling of the brainstem (Kim M. et al., 2022). Thus, when evaluating patients with acute vertigo, clinicians seek to differentiate inner ear conditions from stroke or other central causes (Newman-Toker, 2016). This is a challenging task for the ED clinician because vertigo is the most common posterior circulation ischemic stroke symptom and is frequently unaccompanied by more obvious neurological symptoms (Paul et al., 2013). Overall, dizziness and vertigo are the symptoms most tightly linked to missed stroke (Paul et al., 2013).

### 1.2. Causes of diagnostic errors in emergency department

Acute dizziness in the ED accounts for approximately 4% of visits in the United States (US) (Newman-Toker et al., 2008). Neuroimaging is still considered the gold standard diagnostic test for stroke. However, Computed Tomography (CT) has very low sensitivity for stroke (approximately 16% in the first 24 h after stroke onset) so it is of little use for identifying acute ischemic strokes, particularly in the posterior fossa (Chalela et al., 2007; Edlow et al., 2023). Despite this, nearly 50% of US ED patients presenting with dizziness undergo CT imaging (Saber Tehrani et al., 2013). Even diffusion-weighted magnetic resonance imaging (DW- MRI), considered the gold standard for acute stroke diagnosis, misses up to 20% of acute posterior circulation strokes if performed < 24 h from symptom onset (Newman-Toker, 2016). Requesting neuro-imaging to detect cerebrovascular cases amongst all patients with acute vertigo, most of whom will not have a stroke, presents a substantial challenge with high cost and resource implications (Saber Tehrani et al., 2013). Of the acute dizziness/vertigo cases in ED, an estimated 40% of underlying strokes are missed, even though relatively simple, non-invasive, bedside physical examination tests (such as HINTSplus–Head Impulse Nystagmus Test of Skew plus test of hearing), performed by trained specialists, have been shown to identify more than 99% of strokes (Kattah et al., 2009). Less attention has been given to the use of a focused neurological assessment to “screen out” central neurological causes of acute vertigo that is of course inexpensive.

In this review, we will highlight the importance of a general neurological examination to explore central neurological causes (e.g., stroke) and the use of specific, targeted bedside eye movement examinations (such as HINTS, and Dix Hallpike) to differentiate peripheral vs. central causes of vertigo when nystagmus is present. We describe practical implementation considerations for evaluating patients with acute vertigo, including the timing of diagnostic testing within the ED pathway, testing resources and pitfalls associated with current practices.

### 1.3. Stroke protocols in the emergency setting

The pathway for a patient with suspected stroke is well established by the National Institutes of Neurological Disorders and Stroke (NINDS) (Jauch et al., 2013). Early triage includes obtaining as accurate a patient history as possible, including establishing the last known well time, or time of stroke symptom onset. This information is essential in determining whether an acute stroke patient is a candidate to receive IV tPA (Song, 2013). Positive outcomes on a triage can trigger a seamless process that once in motion offers the patient a treatment within a short window. Triages of standardized stroke assessment tools include the LAPSS [Los Angeles Prehospital Stroke Screen (Kidwell et al., 2000)], the CPSS [Cincinnati Prehospital Stroke Scale (Zohrevandi et al., 2015)] and the FAST score [facial droop, arm drift, speech problem (Dombrowski et al., 2013)], and have been shown to provide consistency in accurately identifying potential strokes (Song, 2013). Triggering a suspected stroke protocol allows each potential stroke patient to be treated with the priority and urgency, given the limited time window of stroke therapeutics. However, acute vertigo (with or without hearing loss) does not typically trigger a stroke protocol. Without a well-established acute vertigo protocol within the ED, the pathway of the acute vertigo patient is far more challenging given that a proportion of these patients will indeed have a posterior circulation stroke.

### 1.4. Differential diagnosis of acute vertigo in the emergency department–clinical history

Differentiating between central (brain) vs. peripheral (ear) causes is key to appropriate initiation of a standardized stroke pathway deployment. As up to 95% of ED patients with dizziness may not have stroke, inappropriate triage to stroke pathways will add to resource burden. No formal established International acute dizzy protocol exists and therefore one approach to dizziness within EDs is to employ the TiTrATE algorithm- that is, timing, triggers and targeted examinations (Newman-Toker and Edlow, 2015; Ali et al., 2018). The TiTrATE algorithm helps determine the evolution of dizziness symptoms into spontaneous or episodic, positional, or spontaneous and derives examinations that can assist in the differentiation of the causes of dizziness (Newman-Toker and Edlow, 2015). The dedicated examination is primarily reliant on bedside eye movement assessments, which we will describe in greater detail, alongside testing considerations that may impact the success of their deployment. Such a clinical decision support offers ED clinicians with varying levels of experience a structured approach to evaluating acute dizziness. This quick evaluation helps differentiate between peripheral and central balance disorders assisting in resource utilization by identifying cases that may not require extensive testing or hospitalization. However, there are limitations to this approach given the need for the clinician to be trained and have familiarity with its components, to ensure consistent and pertinent application.

A recent Barany Society classification document outlined the potential evolution of what they term vascular vertigo, with acute prolonged vertigo refers to symptoms lasting 24 h or more, transient vascular vertigo where symptoms last <24 h but there is imaging confirmation of ischaemia or hemorrhage, and acute vascular vertigo in evolution, where symptoms have occurred for more than 3 h but has not yet lasted for at least 24 h at the time of clinical assessment (Kim J. et al., 2022). In the latter case, patients must have at least one of: focal central neurological symptoms and signs, or severe truncal ataxia/postural instability, at least one component of central HINTS, other central ocular motor abnormalities (e.g., central nystagmus, impaired saccades or impaired smooth pursuit), a new onset of moderate to severe cranio-cervical pain, increased risk for vascular events, (e.g., ABCD2 score of 4 or more, or atrial fibrillation), or significant (> 50%) narrowing of an artery of the vertebrobasilar system on imaging. In transient or evolving scenarios of vertigo, perfusion imaging may be of relevance to help identify a vascular etiology (van der Hoeven et al., 2015).

### 1.5. Diagnostic eye movement examination for dizziness patients in the emergency department

Emergency clinicians are trained to triage urgent patients with the aim to quickly identify critical illness and preserve life. The diagnosis of conditions such as vestibular neuritis, benign paroxysmal positional vertigo (BPPV) and other peripheral vestibular disorders is arguably of lesser priority for the emergency clinician than identification of potentially sinister disorders such as stroke or brain tumors. Discharging an acutely dizzy patient without a confirmed or working diagnosis can have life-altering consequences, however. Most emergency clinicians recognize the importance of bedside eye movement examination when a patient reports acute vertigo and may also perform bedside testing such as Dix-Hallpike and HINTSplus. However, less clear is the degree of training they may have received to perform and interpret such assessments accurately.

When present, nystagmus characteristics can help differentiate between central and peripheral causes of acute vertigo. Peripheral patterns of nystagmus include a spontaneous horizontal/torsional nystagmus enhanced by removal of fixation (https://www.youtube.com/watch?v=eGcUTfeHvZg) and following Alexanders' Law (Kaski and Seemungal, 2010). Central patterns of nystagmus include vertical spontaneous nystagmus and direction-changing (“gaze-evoked”) nystagmus (Kaski and Seemungal, 2010). Eye movement examination requires experience to observe the small intricate eye movements that can result from the pathologies causing nystagmus.

In the ED, HINTS was introduced in 2009 (and later HINTSplus, which added a test of acute hearing loss) as a clinical decision-making tool capable of differentiating between peripheral (e.g., vestibular neuritis) and central (e.g., stroke) vestibular disorders (Table 1) (Newman-Toker et al., 2015). The combination of a positive head impulse test, direction-fixed horizontal or torsional nystagmus, a negative test of skew and the absence of any new unilateral/asymmetrical hearing deficit suggests a peripheral vestibular disorder as the likely cause (Kattah et al., 2009). On the other hand, a negative head impulse test, direction-changing or vertical nystagmus, and a positive test of skew (or new hearing loss together with a positive head impulse test, direction-fixed horizontal or torsional nystagmus) raise suspicion for a central nervous system disorder, such as a posterior circulation stroke (Kattah et al., 2009). HINTSplus stroke sensitivity was reported as 97%, specificity was 84% (Newman-Toker et al., 2013) but only when performed by neuro-ophthalmology/otology specialists, within a more controlled environment. Performing the HINTSplus examination in the ED is challenging–even for specialists; some of the major issues include suboptimal testing environments such as external distractions in a busy environment, inadequate lighting, poor patient compliance, and time constraints that negatively affect the detection of subtle eye movements findings. It is perhaps unsurprising then that many strokes presenting with vertigo are misdiagnosed (Newman-Toker et al., 2022).

**Table 1:** The components of HINTS-plus assessment in the identification central vs. peripheral etiologies in a patient with acute vertigo.

	**Central**	**Peripheral**
Head impulse test	Normal	Abnormal unilaterally
Nystagmus	Direction-changing or pure vertical	Unidirectional (horizontal/torsional)
Test of skew	Large skew deviation (>3 deg)	No, or small (< 3 deg) skew
Plus test of hearing	New unilateral hearing loss	Normal hearing

The Dix-Hallpike maneuver is important eye movement assessment in patients with acute positional vertigo without spontaneous nystagmus, for identification of BPPV, one of the commonest causes of vertigo in the general population (von Brevern et al., 2007). The Dix-Hallpike maneuver is still substantially under-performed in general practice and emergency settings, where most patients with BPPV present (Tahtis et al., 2021). Maneuvers for right and left posterior semicircular canals are performed initially as these are the most involved canals (up to 95% of all BPPV cases) (Parnes et al., 2003). The maneuver takes the sitting patient through a 45-degree head turn followed by a decline to the head hanging over the end of the bed supported by the individual performing the test. In a patient with BPPV, free-floating otoconia, when testing the affected side, results in vertigo together with an upbeat/torsional nystagmus beating to the lowermost ear, that is used to confirm the condition (Parnes et al., 2003; von Brevern et al., 2007). In the absence of a positive Dix-Hallpike, the emergency clinician cannot conclusively diagnose and discharge the patient with a BPPV. BPPV assessment in the acute setting results in many challenges to the clinician including inadequate patient positioning of the head, not reclining rapidly or fully, and lack of training in the angle of head extension. Incorrect interpretation may result from wrong observation of the eye movements (failure to open the eyelids to see the eyes, the patient moving the eyes during the test etc.), or a failure to accurately identify nystagmus or misinterpret random eye movements (when a patient is moving their eyes around) as pathological.

### 1.6. Pitfalls of diagnostic eye movement examination

Bedside eye movement examination, the Dix-Hallpike and the HINTSplus all require a reasonable level of cooperation from the patient that cannot be assumed when testing a patient with hyperacute vertigo. Non-specialists with minimal training may need longer to examine and identify any eye movement abnormality (Tarnutzer et al., 2023). Assessment techniques that follow a standardized approach can be highly effective, such as the Dix-Hallpike for BPPV diagnosis (von Brevern et al., 2015). Thus, some of the pitfalls of ocular motor assessment can be mitigated through improved environment setup, such as selecting assessment areas that do not have external distractions, better lighting for observation of eye movements, and adapting maneuvers to less convenient settings [e.g., side-lying Dix-Hallpike (Kaski and Bronstein, 2014)]. Gaining proficiency by using a standardized technique, through familiarity and specialized training, could also lead to improved diagnostic evaluation. Presently, ED clinicians do not receive specialist training in eye movement assessment, although many recognize it would be advantageous (Warner et al., 2022).

**Figure 1:**
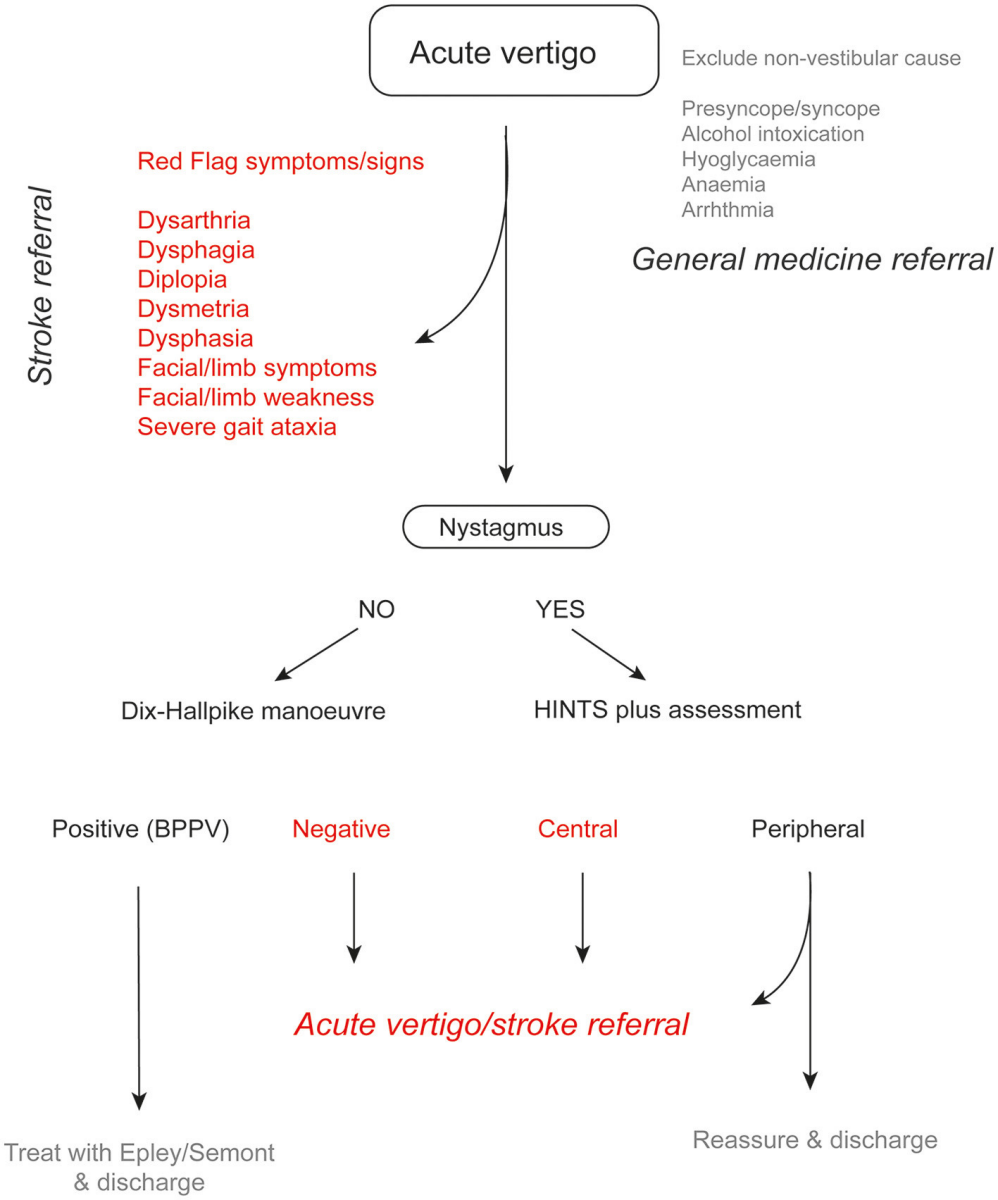
Suggested algorithm in a patient with acute vertigo. In patients with an acute vestibular syndrome, HINTS plus assessment can help identify patients with a peripheral etiology (e.g., vestibular neuritis). In such cases, patients can be reassured and discharged once symptoms have improved. If there are any atypical features (headache, neck pain etc.), referral to an acute vertigo team is recommended.

It is worth noting that in one study, the vast majority (~72%) of patients with posterior circulation stroke had motor deficits, 19% had vertigo, and 12% had nystagmus (Tao et al., 2012). This indicates that algorithms focusing on nystagmus assessment are only relevant for a minority of patients with posterior circulation stroke and highlights the importance of conducting a general neurological examination in all patients first, including assessment of gait and truncal ataxia (Figure 1) (Carmona et al., 2023). Other features that should be explicitly sought including focal weakness or paraesthesia of the limbs or face, dysarthria, diplopia, dysphagia, or dysphonia.

### 1.7. Technology and education to improve acute vertigo assessment

One obvious approach to improving correct diagnosis of the patient with acute vertigo is through optimizing the expertise of ED clinicians. Challenges with education include staff turnover in the ED, restricted time for education, being under-resourced, and the need for repeated training sessions given the breadth of disorders ED clinicians deal with. Below we explore some of the interventional options to overcome the diagnostic assessment pitfalls.

#### 1.7.1. Digital trends

Since the onset of the COVID-19 pandemic, several digital macro trends have accelerated and gained prominence, forcing remote working and collaborations online. Organizations rapidly adopted digital tools and technologies to adapt, including digital platforms. The infrastructure and tools to support this digital world has advanced. Tele-health/virtual care witnessed a significant shift toward remote services and e-learning. The adoption of consultations in telemedicine to minimize in-person visits and ensure patient safety gained widespread acceptance and has continued post-pandemic. Cloud computing services surged as organizations sought scalable and flexible infrastructure to support digital operations. These trends have reshaped the way we work, learn, access healthcare, and interact leading to long-lasting changes in our digital behaviors and preferences.

#### 1.7.2. Digital educational platforms

Digital platforms offer easy access to medical educational resources regardless of geographical location creating accessibility to all. These digital platforms allow flexibility in schedules, as physicians are exceptionally busy, work shifts, and have other commitments. The benefits of access 24/7 bring convenience and greater user interaction. There is the possibility to display a library of eye movements and the “type of nystagmus” labeled that can transfer better identification in “real life” settings (Tahtis et al., 2021). Quality of procedures can be standardized and updated in real time especially as education within the acute dizzy field is evolving and advancing. Clinicians can be updated on current and relevant materials to support their knowledge and staff turnover can be accommodated in a timelier manner. Overall, a digital medical education platform offers convenience, flexibility, interactivity, and personalized learning experiences, enabling students and professionals to access high-quality medical education resources anytime, anywhere.

#### 1.7.3. Telemedicine and virtual care

Telemedicine offers several benefits to diagnostics in the ED. It allows access to real-time experts who do not need to be present within the facility promoting time savings and rapid assessments. Remote assessments require eye recording technology to aid the eye assessment in the patient with acute vertigo however does not solve the issue that a trained clinician is required for bedside maneuvers (Dix-Hallpike and HINTS). Virtual care can save crucial time in the diagnostic process, enabling faster decision-making and initiating appropriate treatments promptly. This is particularly beneficial for hospitals in rural or underserved areas where training and specialists may be limited. Rapid triaging of patients to discharge or determining the need for specialized interventions allows ED clinicians to make informed treatment decisions and optimize patient outcomes. This can help reduce unnecessary patient transfers from smaller or rural hospitals to larger stroke centers reducing transfer rates that overcrowd and burden stroke centers.

Wilcock and colleagues found that TeleStroke hospitals had higher rates of reperfusion treatment compared with those cared for at control hospitals (Wilcock et al., 2021). TeleStroke networks have been deemed to be cost-effective when compared with usual care in cost-effectiveness analysis (Nelson et al., 2011). The reality is a substantial outright investment is needed to implement TeleStroke networks; however, the return from savings is on eliminating the need for additional on-site specialists and potentially reducing staffing costs. Telemedicine in acute vertigo diagnostics could enhance the speed, accuracy, and accessibility of specialized care in the ED. In the UK however, the lack of neuro-otology specialists (and other acute vertigo specialists) would render a Telemedicine approach for acute vertigo difficult to scale, beyond a few major city university teaching hospitals.

#### 1.7.4. Technology to assist clinical expertise

Eye recording technology to capture and analyse eye movements can aid the interpretation of any nystagmus in patients with acute vertigo. Video recording (with expensive oculography or more accessible smartphone technology) can assist in the identification of subtleties of eye movements as the recordings can be played back at slower speeds. Advancements in video goggles to include a gyroscope, and accelerometer measurements can also aid in the quality and standardization of assessments such as HINTSplus and the Dix-Hallpike maneuvers. Further advancements may include expansions in algorithms and artificial intelligence in medical practice that could offer triaging or screening in this setting, offering enhanced diagnostic consistency and standardized care (Wagle et al., 2022; Kong et al., 2023; Wu et al., 2023).

### 1.8. Acute vertigo protocols

Hyperacute assessment of the dizzy patient represents, in our experience, the greatest clinical challenge in a patient with acute vertigo (Kaski et al., 2023). Indeed, due to the associated symptoms of nausea, vomiting and lack of patient cooperation it is common for bedside eye movements and gait assessment to be performed once the patient stabilizes. Timing of patient assessment is imperative to the outcome for the patient with anterior circulation stroke (Saver, 2006). However, there is a lack of guidance regarding whether a patient with isolated acute vertigo (and hearing loss) fulfilling a HINTS “central” outcome, should receive thrombolysis when presenting within the therapeutic time window. The current criteria rely partly on a burden of presenting symptoms (NIH stroke scale), this scale does not include vertigo or hearing loss (Chalos et al., 2020). However, the presentation of posterior circulation stroke is less common than a peripheral cause, only accounting for 10-20% of all acute vestibular syndromes (Tarnutzer et al., 2011). Most patients with *possible* posterior circulation stroke do not receive thrombolysis in the ED or hyperacute stroke unit settings because patients often improve substantially following isolated vestibular stroke syndromes (without significant cerebellar involvement). It is less clear whether the same is true for acute hearing loss in the context of a posterior circulation stroke. The posterior circulation provides blood flow to the inner ear, and mixed audiovestibular presentations have been reported in patients with cerebrovascular disease [3]. Although less common, hearing loss accompanied with acute vestibular syndrome can be caused by an ischemic disturbance of the inner ear, which is often associated with an infarction of the anterior inferior cerebellar artery (posterior circulation territory). Provision of a protocol that recognizes that preservation of life and “brain” is paramount above all and suggesting closer monitoring in the 24–48 h post initial presentation of symptoms would standardize care for patients with acute vertigo. Such guidance might recommend that a secondary prevention measure is instigated in patients with possible posterior circulation stroke, and appropriate investigations performed (e.g., vascular imaging for vertebral artery dissection).

## 2. Conclusions

Diagnosing patients with acute vertigo in the ED, particularly in detecting strokes, poses significant challenges that can lead to diagnostic errors and delayed treatment. By implementing standardized protocols, closer monitoring, and utilizing advanced diagnostic tools, healthcare professionals can mitigate diagnostic errors, provide timely and accurate assessments of the patient with hyperacute vertigo, and ultimately optimize outcomes in the ED.

## Author contributions

SM: Conceptualization, Validation, Writing—original draft. NK: Conceptualization, Validation, Writing—review and editing. DK: Conceptualization, Methodology, Project administration, Resources, Supervision, Validation, Writing—original draft, Writing—review and editing.
